# Blockchain support for execution, monitoring and discovery of inter-organizational business processes

**DOI:** 10.7717/peerj-cs.731

**Published:** 2021-09-29

**Authors:** Miguel Morales-Sandoval, José A. Molina, Heidy M. Marin-Castro, Jose Luis Gonzalez-Compean

**Affiliations:** 1 Unidad Tamaulipas, Centro de Investigacion y de Estudios Avanzados, Victoria, Tamaulipas, Mexico; 2 Facultad de Ingeniería y Ciencias, Cátedras-CONACYT, Universidad Autónoma de Tamaulipas, Victoria, Tamaulipas, México

**Keywords:** Blockchain, IOBP, Process mining, Execution, Monitoring, Discovery

## Abstract

In an Inter-Organizational Business Process (IOBP), independent organizations (collaborators) exchange messages to perform business transactions. With process mining, the collaborators could know what they are actually doing from process execution data and take actions for improving the underlying business process. However, process mining assumes that the knowledge of the entire process is available, something that is difficult to achieve in IOBPs since process execution data generally is not shared among the collaborating entities due to regulations and confidentiality policies (exposure of customers’ data or business secrets). Additionally, there is an inherently lack-of-trust problem in IOBP as the collaborators are mutually untrusted and executed IOBP can be subject to dispute on counterfeiting actions. Recently, Blockchain has been suggested for IOBP execution management to mitigate the lack-of-trust problem. Independently, some works have suggested the use of Blockchain to support process mining tasks. In this paper, we study and address the problem of IOBP mining whose management and execution is supported by Blockchain. As contribution, we present an approach that takes advantage of Blockchain capabilities to tackle, at the same time, the lack-of-trust problem (management and execution) and confident execution data collection for process mining (discovery and conformance) of IOBPs. We present a method that (*i*) ensures the business rules for the correct execution and monitoring of the IOBP by collaborators, (*ii*) creates the event log, with data cleaning integrated, at the time the IOBP executes, and (*iii*) produces useful event log in XES and CSV format for the discovery and conformance checking tasks in process mining. By a set of experiments on real IOBPs, we validate our method and evaluate its impact in the resulting discovered models (fitness and precision metrics). Results revealed the effectiveness of our method to cope with both the lack-of-trust problem in IOBPs at the time that contributes to collect the data for process mining. Our method was implemented as a software tool available to the community as open-source code.

## Introduction

An Inter-Organizational Business Process (IOBP) is an organized group of joined activities carried out by two or more organizations to achieve a common business objective (Bouchbout & Alimazighi, 2011). In recent years, digital revolution has promoted the digitalization of IOBPs for competitive and for satisfactory customer experience. In this context, organizations are, for example, creating IOBP models represented digitally under BPMN (Business Process Model and Notation) or using Business Process Management (BPM) workflow engines to perform a complete orchestration of all process flows. Information systems supporting process digitalization contribute to make processes more efficient while serve as a medium to obtain and record detailed process execution data.

Process improvement is a need for organizations to ensure quality of service and to obtain greater customer satisfaction. First, organizations need to gain a profound understanding of the process, that is, to uncover the real behavior of their executed process to then identify process improvement opportunities. This can be achieved from data already collected by process’ information systems.

By extracting knowledge from events produced by the process execution, process mining (PM) (van der Aalst & Weijters, 2004; van der Aalst, 2012) is a powerful tool to gather insights in how a given process actually executes. The behavior observed in the business process is revealed from models automatically constructed from the event logs. Although most of the process mining techniques were firstly conceived and applied for intra-organizational process, that is, a process under the control of a single organization, those techniques have also been extended for IOBPs (van der Aalst, 2011; Engel et al., 2012; Engel et al., 2016).

Different to intra-organizational business processes, where all the involved parties in the process are trusted, in an IOBP the control is handled collaboratively by the participants, generally from different untrusted organizations. This results in an inconsistent and untrusted process management (Nakamura, Miyamoto & Kudo, 2018) as in regular IOBP there is lack of full knowledge by the participants on the status of the IOBP’s tasks being conducted. Furthermore, the IOBP participants may blame each other (Sturm et al., 2019) when disputes arise in such a collaborative process. For that reason collaborators need relying on authorized third parties to mediate and control the execution of IOBPs. However, using a trusted third party implies a centralized control which imposes other restrictions for the participants, increases exchange of messages, and the executed processes can still be subject to dispute on counterfeiting actions from participants, inclusive by third parties (Claudio Di Ciccio et al., 2019).

The problem posed by the lack of mutual trust in IOBP execution has been proposed to be managed by using Blockchain technology (Weber et al., 2016; Mendling et al., 2018; Carminati, Ferrari & Rondanini, 2018). Blockchain (Casino, Dasaklis & Patsakis, 2019) can be viewed as an abstract data structure, that is, with well-defined operations. This data structure is a list of linked blocks comprising transactions in a certain domain, for example, payments. Operations on this data structure are executed by a peer-to-peer (P2P) network of participants (full nodes); each node holds the entire Blockchain locally. Every new transaction is broadcasted within the P2P network, where each node adds it to its pool of unconfirmed transactions. At some moment, a full node will try to create a new block (set of transactions) for the Blockchain. Only the node that solves a cryptographic puzzle can add the block to the Blockchain, and all the unconfirmed transactions in that new block will be removed from the pool of all other nodes. The node adding the new block to the Blockchain receives the fees associated to the transactions in the block. A new propagated block in the P2P network can be double checked against a set of specific rules before being added permanently to the Blockchain.

A smart contract is a user-defined program executed on the Blockchain network (Macrinici, Cartofeanu & Gao, 2018). Smart contracts are distributed to all full nodes of the network, so their use must be carefully on the side of security and privacy of shared data managed by the program. Transactions in the Blockchain are the result of calling smart contract functions.

Blockchain and smart contracts are disruptive technologies impacting in several fields such as in agriculture (Pranto et al., 2021), health (Sookhak et al., 2021), automotive (Narbayeva et al., 2020), energy (Andoni et al., 2019) and business process management (Mendling et al., 2018), to mention a few.

Thus, in the context of IOBPs and process mining, instead of agreeing on one trusted party, collaborators in the IOBP share transactional data related to the IOBP execution in a Blockchain, which ensures the interactions conform to the IOBP choreography model. A smart contract is used as a direct implementation of the mediator process control logic and trust is achieved due to Blockchain’s consensus mechanisms.

Few works have studied process mining aspects of business process supported by Blockchain, but as a separate problem. For example, some works assume that process execution data is already on the Blockchain (Mühlberger et al., 2019; Klinkmüller et al., 2019; Duchmann & Koschmider, 2019). Under that assumption, meaningful process data is extracted from blocks, mapped to an event log model, and finally transformed into an event log for later processing of process mining algorithms. Other works, as in Ekici, Tarhan & Ozsoy (2019), focused on data cleaning of events retrieved from Blockchain transactions to form a useful event log. To the best of our knowledge, no one of the previous works consider, at the same time, issues regarding management, monitoring and process mining of IOBP. We hypothesize that these issues can be jointly addressed as the event log required for process mining can be obtained at the time the IOPB is being trustingly executed. Thus, this work aims at answering the following research question: *Is it possible to construct a method that ensures the correct execution and monitoring of inter-organizational business processes at the time that it includes an strategy for collecting and preparing the associated event data, useful for process mining tasks?*

Our goal is to provide a practical solution to integrate Blockchain and smart contracts technologies not only for supporting IOBP execution management but also for IOBPs process mining. To achieve this goal, we present as contribution a method that takes advantage of Blockchain capabilities to tackle, at the same time, the lack-of-trust problem in IOBPs (monitoring and execution) and the IOPB mining (event log collection). We take advantage of the logic required for process management to collect, with dynamic data cleaning on-chain, the event log that can be later used for process mining. Our proposed method, comprised of four main algorithms, is realized as a software tool. The first algorithm takes an IOBP choreography model as input, in the BPMN notation, and configures a smart contract that ensures the control flow and business logic implied by the IOBP. Once configured, the smart contract orchestrates each IOBP instance execution according to the documented model. Collaborators are modeled as triggers that interact with the smart contract through web services. While the IOBP executes, the second algorithm collects the process events to dynamically create the event log (on-chain). On the fly, a third algorithm performs data cleaning over the events from triggers to ensure a more accurate event log. A fourth algorithm creates the event log in standard format (XES or CSV) to be used as the input model in process mining tools for IOBP discovery and then evaluate conformance. In this work, process mining tasks are performed using Prom (van Dongen et al., 2005) and P-Miner (Marin-Castro & Tello-Leal, 2021) tools.

We implemented our method as a software tool and use it to evaluate our approach by executing a series of experiments for three IOBPs. Experiments comprised (*i*) the setup of the smart contract for monitoring the IOBP execution, (*ii*) the execution of several IOBP instances to create an event log, (*iii*) formatting the event log (XES and CSV) for PM, and (*iv*) obtaining the IOBP models from PM tools (Prom and P-Miner). For evaluation purposes, we simulate noise generation to model typical errors in the events generated from the collaborators and present in the event log. We consider the most common cases of noise in the event log (Suriadi et al., 2017; Song et al., 2021). We report details on the experiments and results from the obtained models, on the metrics of fitness, precision, and *F*-measure. The software tool is open-source and available at https://github.com/amolina-97/IOBPBC. Results from the experimentation revealed that our method is effective and suitable for practical use.

The rest of this paper is organized as follows. “Background” presents the preliminaries and background of this research. “Methods” describes the methodology to deploy IOBPs in Blockchain and to perform IOBP mining. “Experimental Evaluation and Results” presents the experimental evaluation and results obtained from the process mining tasks. Finally, “Conclusion” concludes this work and provides insights of future work.

## Background

This section summarizes the essential aspects of the two main fields in this research: Blockchain technology and process mining.

### Blockchain and smart contracts

Blockchain can be seen as a distributed data storage structure. However, it is a very interesting data structure different to other widely known such as linked lists. Blockchain includes mechanisms that provide security services on the data being stored, for example, integrity, authenticity and tamper-resistance. Smart contracts are basically programs executed on top of Blockchain. Thus, they inherit security properties of the Blockchain, such as integrity, authenticity and immutability. The Ethereum Blockchain is one of the blockchains most used in practice (Oliva, Hassan & Jiang, 2020). Ethereum includes an Ethereum Virtual Machine (EVM) which is in charge of executing smart contracts.

To execute a smart contract in an Ethereum Blockchain, it must be first deployed in the blockchain by means of a transaction. Any transaction in the Blockchain requires a digital wallet (Dwyer, 2015), an artifact that allows users to make electronic transactions. Digital wallets are constructed from public key cryptographic material to authenticate the user and the user’s transactions. The user signs its transactions with a private key and any other entity can verify the authenticity of such transaction using the user’s public key, which is mathematically associated to the private one.

**Figure 1:**
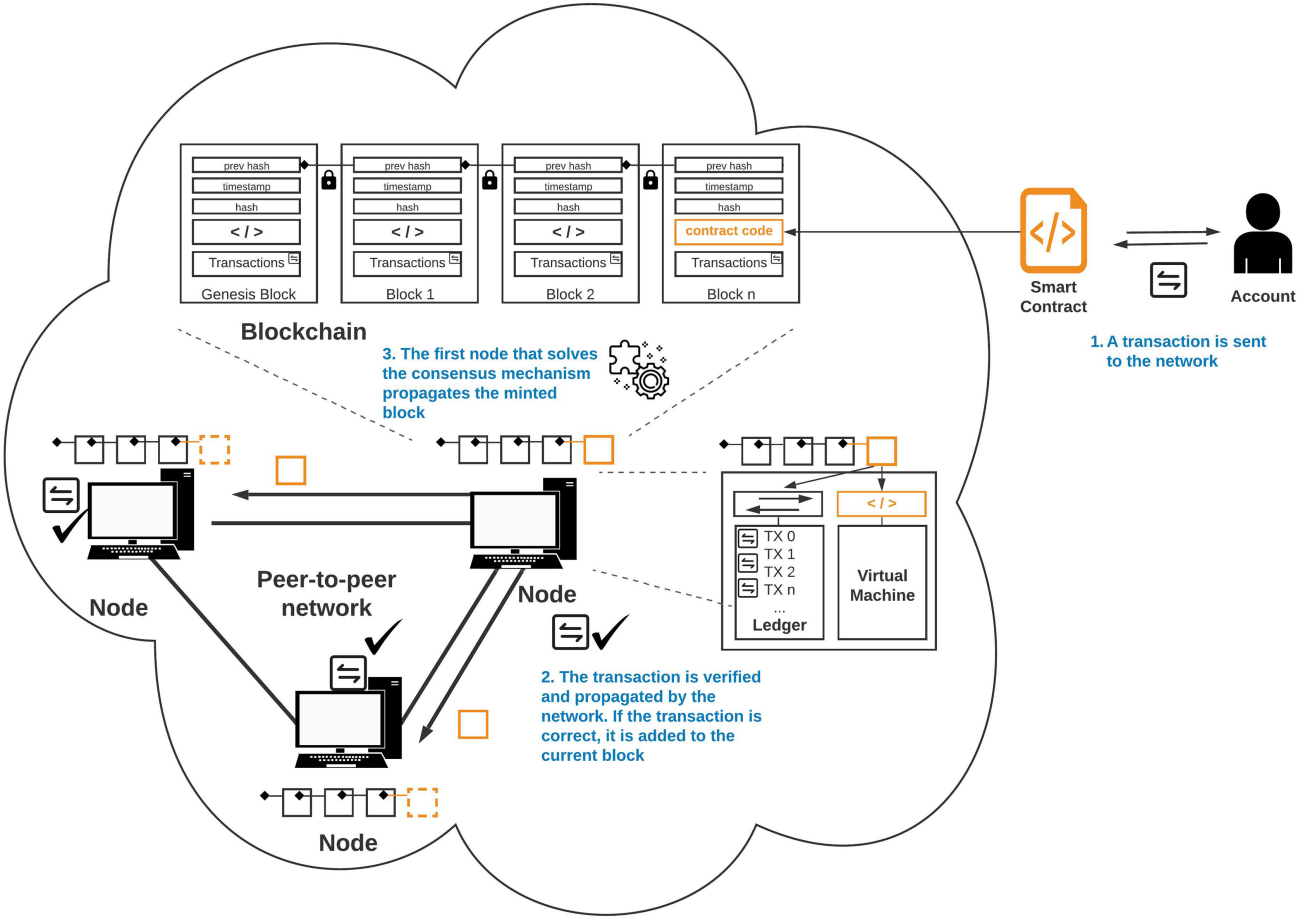
Interaction between Blockchain and smart contracts.

Thus, the responsible node for deploying the smart contract sends a transaction to the Blockchain. The transaction is verified and validated in accordance to the Ethereum protocol. The execution of this transaction deploys the smart contract in the EVM, in each node in the P2P network. The code of the smart contract is stored permanently in the Blockchain for future invocations. Once deployed, the smart contract is assigned with an address that any user can invoke to interact with the code associated to the smart contract. When a new transaction comes to the Blockchain containing the smart contract address, all the miner nodes in the P2P network execute the smart contract with the current state of the Blockchain and with the same input parameters in the transaction. Figure 1 graphically shows the interaction between a Blockchain and the smart contracts previously described.

### Process mining

Process mining (van der Aalst, 2012) is a research area where converges machine learning, data mining and analysis and modeling of processes. A business process model can be represented as a directed connected graph *M* = {*N*, *E*_*m*_}. *N* is the tuple {*i*, *o*, *T*, *G* ^+^ , *G*^*x*^, *G*^*o*^}, with *i* the initial event, *o* the end event, and *T* the set of tasks executed in the process. The flow of tasks is controlled by gates. *G*^+^ is the set of AND gates, *G*^*x*^ is the set of XOR gates and *G*^*o*^ is the set of OR gates. *E*_*m*_ is the set or arcs in the graph of the form (*a*; *b*), with {*a*, *b*} ∈ *N*. An XOR gate creates alternative execution paths, but only one path can be followed. An AND gate divides an execution path in parallel paths. An OR gate indicates that one or more execution paths can be followed. Processes are supported by information systems. Each event during the execution of an instance process can be registered and the set of all these events is a trace.

The set of all traces conform the event log, which is the main data for process mining. Three types of tasks can be executed in process mining (van der Aalst, 2012): process models discovery, conformance checking and enhancement. Discovery consists in using the event log to create a model *M′* that explains the real behavior of the process, which generally is not the same as the expected documented model *M*. Conformance consists in comparing the discovered model with its event log and measuring alignment. That comparison is done using quality dimensions such as fitness and precision. Conformance can be used check whether the reality registered in the event log adjusts to the process model and vice verse. Finally, enhancement uses the model to identify improvement opportunities in the process when executed.

## Related work

This section discusses representative works related to this research. For the sake of clarity, we first discuss approaches for management and monitoring of business process using Blockchain. Then, we discuss on works for process mining using data from Blockchain. This review frame the originality and contributions of our approach.

### Blockchain for business process execution and management

Weber et al. (2016) were pioneers in suggesting the usage of Blockchain for business process management (BPM), particularly for execution and monitoring of IOBPs. Blockchains were proposed to execute processes in a trustworthy manner even without a mutual trust between collaborators. They proposed transforming large parts of the control flow and business logic of an IOBP into smart contracts ensuring that the IOBP is correctly executed. Trigger components were proposed to connect the IOBP implementation in the smart contract to internal process implementations, that is, triggers served as a bridge between the Blockchain and collaborators’ applications. A trigger runs in a full node of the Blockchain network.

A smart contract is used as a direct implementation of process control logic mediator. The smart contract, one per process model, checks if collaborators’ interactions are conforming to the IOBP choreography model. A translator takes a business process specification as input and creates artifacts (at design time) for the triggers and the smart contract to collaborate each other over the Blockchain network. Those artifacts (factory contract) are later configured when the IOBP is instantiated and executed. Artifacts include an interface specification per collaborator in the IOBP to be distributed to the respective triggers and a process instance contract deployed on the Blockchain when the process is instantiated. The process instance contract contains the implementation of the IOBP business logic.

For each new process instance, the participants register their roles and public keys (wallet address of a collaborator in the IOBP). These roles and keys are available to all triggers associated with the process instance. When the process instance is executed, each trigger receives both API calls from its owner and logical messages from the process instance contract. The trigger translates each of its API calls (messages) into a Blockchain transaction. In case receiving a logical message from the instance contract, the trigger updates its local state and invokes the execution of logic for the collaborator’s private process. The seminal work by Weber et al. (2016) has served as the basis for implementing systems for Blockchain-based process execution and monitoring, such as the Caterpillar and Lorikeet systems (Claudio Di Ciccio et al., 2019).

Later, Sturm et al. (2019) presented an architecture that contrary to the approach by Weber et al. (2016), uses a single generic Smart Contract to deploy in the Blockchain the execution and management of any IOBP. In their proposal, authors do not generate a Smart Contract (no programming code is generated) from an IOBP model but a generic Smart Contract is filled with logic by transactions at deployment time. The generic Smart Contract is first configured by means of specific functions calls (transactions) that allow the setup of collaborators, IOBP tasks and the IOBP logic.

Each IOBP collaborator has a unique identifier (wallet) that allows it to join the Blockchain network for executing an IOBP task. First, a supervisor responsible for the initial setup deploys the Smart Contract and adds collaborators by registering their wallets. Then, the supervisor implements the IOBP logic by using a data structure that models an IOBP task. If viewed as a directed graph, a task in the IOBP can only be executed if its incident tasks (requirements) have been completed (conformance system). In case of gateways, the logic is implemented based on the gateway type. In case of the parallel gateway, all the incident task must be set on completed. The Smart Contract ensures that each task in the IOBP is executed only by the collaborator in charge of such task (permission system). Thus, the IOBP logic is built based on the conformance and permissions systems. Contrary to the approach in Weber et al. (2016), where the process logic is defined off-chain, in Sturm et al. (2019) the IOBP logic is also on-chain so every collaborator can comprehend the IOBP rules.

In this paper, we retake the idea of Sturm et al. (2019) to have a generic Smart Contract applied to any IOBP (1:*M* relation). However, we extend that approach to consider the following:

-Support is not only for OR and AND gateways but also for XOR (exclusive) gateways.
-Support for executing multiple IOBP instances in a single Smart Contract instance.
-Inclusion of Trigger component (as in Weber et al. (2016)) to enable external resources to interact directly with the Blockchain.
-Support to use event information from collaborators to conform the event log required for process mining tasks.

The last property is very important because neither Weber et al. (2016) nor Sturm et al. (2019) approaches the process mining (PM) problem in IOBPs. Both works do not take into consideration the logic for collecting the event log as part of the execution and monitoring tasks. Particular differences of the settings in this paper for IOBP execution and monitoring (E & M) is summarized in Table 1 (SC stands for Smart Contract).

**Table 1:** Approaches to execute and monitoring Blockchain-based IOBPs.

Ref.	BPMN model input	SC code generation	SC per Process model	SC per Process instance	E & M	PM
Weber et al. (2016)	✓	✓	1:1	1:1	✓	✗
Sturm et al. (2019)	✓	✗	1: *M*	1:1	✓	✗
This work	✓	✗	1: *M*	1:*M*	✓	✓

Related works as by Weber et al. (2016) generates specific smart contract code for each IOBP. This is not recommended because a translation process is required, from BPMN specification to code of the smart contract (Solidity for example). However, that translation could not be applicable in all cases. Instead, this work uses a single smart contract, as in Sturm et al. (2019) that only requires configuration at execution time. That is, the code (smart contract) is generated in advance and already running in the Blockchain. Any IOBP can be configured in that unique smart contract, so the smart contract can support any IOPB and only requires configuration by a supervisor node.

Other works in the literature use different approaches to execute and monitoring Blockchain-based business processes. For example, Nakamura, Miyamoto & Kudo (2018) propose transforming business process models into statecharts. The size of these statecharts is optimized to reduce the communication between the Blockchain and collaborators. The statecharts are used as the basis for generating the Smart Contracts to run in the Blockchain and web applications for the collaborators. We consider that this approach is more elaborated and hard to generalize and apply to any IOBP.

### Event log collection from Blockchain data

Process mining of inter-organizational processes (IOBPs) was suggested since the last decade (van der Aalst, 2011). The event log, which is the main input for process mining algorithms, has been proposed to be obtained from Blockchain. That is, in most of related works, it is necessary *an extraction task* on the Blockchain data to build the event log on the basis that the business process execution data was registered in the Blockchain. Klinkmüller et al. (2019) stated that extracting data from Blockchains for analyzing the process view is hard and that is the reason why Blockchain data are rarely used for process mining. They proposed a framework that includes an extractor for retrieving data from Blockchain that is then transformed into event data and formatted according to the XES standard. In that format, the extracted data can be imported into process mining tools such as ProM. The extraction rules are adapted *via* a manifest which specifies how to filter events, which timestamps to include and where to find the activity name.

Mühlberger et al. (2019) also presented an approach to retrieve process data from an Ethereum Blockchain and to convert those data into an event log in XES format that can be used by a process mining tool. They pointed out that manual effort is required to convert data from individual blocks in the Blockchain into an appropriate format for process mining. That task is hard because the information retrieved from the Blockchain is in hexadecimal and numeric formats, timestamps are approximates as the time in Blockchains is at the level of blocks, and the structure of data payloads in the Blockchain transactions is generally arbitrary. Thus, to identify and convert Blockchain data into a format used by process mining tools, it is necessary a profound understanding of the data model represented on the Blockchain.

As previously stated, in this paper we do not implement an extraction task but exploit the fact that a smart contract already exists to monitor the IOBP execution and to ensure that the IOBP rules are meet by collaborators. Thus, we take advantage of that contract and propose additional logic that collects the traces and then creates on-chain the event log from the events received from collaborators during the IOBP execution. In this way, no identification, no extraction and no conversions of data types are required to conform the event log that can be later used for process mining.

Data cleaning for process mining with smart contracts has been previously suggested by Ekici, Tarhan & Ozsoy (2019). In our proposal, the event log is created on-chain, which implies the data is reliable for all those mutual untrusted collaborators that wanted to apply process mining tasks. When generating the traces, a data cleaning task is also done on-chain to repair the events containing common errors (Suriadi et al., 2017), such as duplicate events, errors in timestamps, or events with incomplete data. If the data cleaning were not done on-chain, the event log could be manipulated locally, the resulting data could not be reliable and the discovered models could not reflect the real behavior.

Table 2 summarizes the main aspects in our proposal compared to previous works on process mining of business process supported by Blockchain data and data cleaning using Smart Contracts. As indicated, in our proposal the algorithm (here on referred as the **SC_ELC** smart contract) for collecting the event log works close related to the algorithm (here on referred as the **SC_EM** smart contract) for execution and monitoring, both on-chain. This way data extraction and conversion types from transactions registered in the Blockchain are avoided at the time that an algorithm (here on referred as the **SC_PDC** smart contract) applies data cleaning on the fly. Later in the experiments, we show the impact of the data cleaning strategy in the resulting IOBP discovered models, under the metrics for precision, fitness and F-measure. Note that this desirable evaluation has not been reported in previous works.

**Table 2:** Approaches to create a IOBP event log on-chain for process mining.

Ref.	SC for exec & monitoring	Extraction algorithm & types conversion	Data cleaning	PM test
Klinkmüller et al. (2019)	✗	✓	✗	✓
Mühlberger et al. (2019)	✗	✓	✗	✓
Ekici, Tarhan & Ozsoy (2019)	✗	✗	✓	✗
This work	✓	✗	✓	✓

## Methods

**Figure 2:**
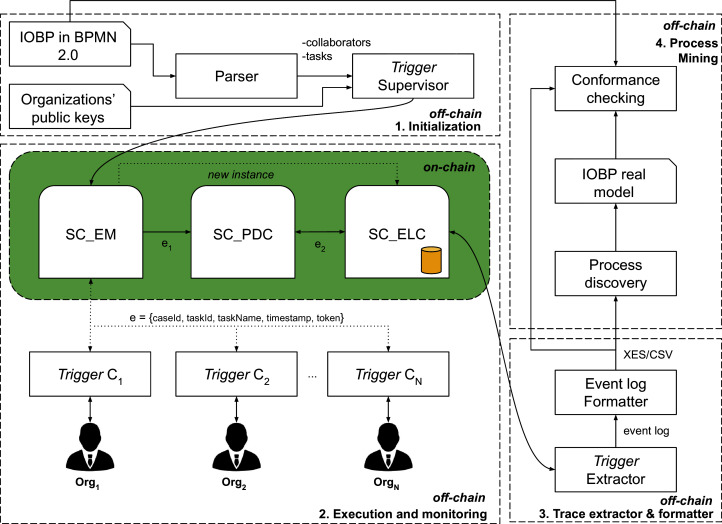
Methodology for monitoring IOBPs execution with Blockchain and for collecting the data, on-chain, required for IOBPs mining.

This section presents the details of our proposed methodology for monitoring the execution of IOBPs with Blockchain and for collecting the data, on-chain, required for IOBPs mining. Such methodology consists in the following chained tasks: (1) Deploy the IOBP in a Blockchain for execution and monitoring, (2) IOBP execution orchestration at the time the event log is collected (on-chain); (3) event log formatting and (4) process mining. This methodology is graphically shown in Fig. 2.

### IOBP deployment in Blockchain

**Figure 3:**
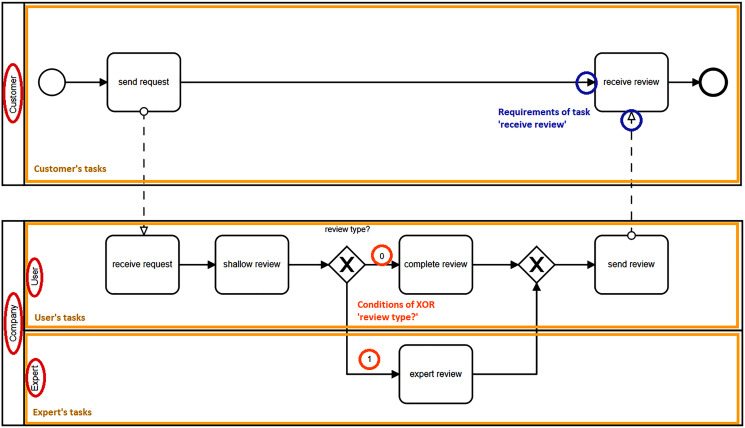
IOBP for a review process.

In the *initialization* stage, a *parser* takes as input the IOBP model specified in BPMN notation, in XML format. The parser extracts the collaborators, tasks and their requirements, gates and their conditions, and the IOBP logic (*e.g.*, communication paths among collaborators). Pools and lanes are considered as collaborators in the input BPMN model. Tasks (activities or gates) and their requirements are identified from each pool or lane in the IOBP model and assigned to the corresponding collaborator. Figure 3 shows an IOBP for a review process. That IOBP has two pools, one for *Customer* and another for *Company*. The last pool has two lanes, one for *Expert* and the other for *User*. Thus, the parser identifies four collaborators in the IOBP.

Task’s requirements are a critical part for ensuring the correct execution of an IOBP. A task *t*_*i*_ owned by collaborator *c* can only be executed if all its incoming adjacent tasks *T*_*i*_ (requirements) have been already successfully executed. Furthermore, if *t*_*i*_ has as requirement an exclusive gate (XOR), the condition associated to that gate must be met. As an example, in Fig. 3, *t*_*i*_ = *‘receive review’* has *T*_*i*_ = {*‘send request’, ‘send review’*} as requirements. For *t*_*i*_ = *‘complete review’*, the requirement is the condition *type = 0*. Conditions are identified by the parser, updated during the IOBP execution, and used as control values in the requirements of outgoing adjacent tasks to the gate. These conditions are available to all collaborators and any of them can modify the condition variable following the rules of the IOBP. The management of XOR gates and associated conditions were not considered in the previous work of Sturm et al. (2019), where only considered {AND, OR} gates that do not require a control of the execution paths.

All the previous data identified by the parser (*c*’s, *t*_*i*_’s, *T*_*i*_’s and condition values) are delivered to a *supervisor* that directly interacts with **SC_EM** to configure it by calling specific functions. This smart contract follows the approach by Sturm et al. (2019), and includes configuration functions such as *addCollaborator(…)* which registers the wallet address (public key) of each collaborator in the IOBP. Another function is *createTask(…)* that registers the activities and gates in the IOBP based on the *requirements* established by the IOBP logic. Contrary to the solution given by Sturm et al. (2019), the initialization phase is executed automatically after receiving the BPMN model and the digital wallets of collaborators. Wallets allow **SC_EM** to receive transactions from collaborators (*e.g.*, by calling a **SC_EM**’s function).

Both, the parser and supervisor implement the deployment of an IOBP in the Blockchain. Algorithm 1 shows the pseudocode that implements the *Initialization* phase shown in Fig. 2.

**Algorithm 1:** Deployment of an IOBP in Blockchain.

**Require:** *iobp*: Inter-organizational business process model in BPMN, *cwallets*: wallets (public key) of organizations in the IOBP, *swallet*: supervisor’s wallet
1: *supervisor*←createTrigger(*swallet*)
2: *config*←Parser.run(*iobp*)
3: *SC_EM* ←*supervisor*.deployContract()
4: **for** *c* in *config*.collaborators **do**
5: *SC_EM*.addCollaborator(*c*.name, *cwallets*.getAddress(*c*.name))
6: **end for**
7: **for** *t* in *config*.tasks **do**
8: *SC_EM*.createTask(*t*.activity, *t*.executor, *t*.type, *t*.requirements, *t*.condition)
9: **end for**

In Algorithm 1, a trigger is created for the supervisor (line 1) to interact with the Blockchain, and in this case, to configure the smart contract based on the IOBP specification in BPMN. The supervisor parses the BPMN specification of the business process and proceeds to deploy and configure the smart contract (lines 2 to 8). Tasks are configured as well as the collaborators responsible to perform each of them. Wallets for the collaborators are registered because each collaborator have to interact with the Blockchain. For a given task, several parameters are specified such as the responsible of that task (executor), the type of task (activity or gate), requirements for that task (preconditions to such task be executed) and condition associated in case the task is a XOR gate.

### IOBPs execution and monitoring

Once **SC_EM** is configured, it is ready to be executed by interacting with the triggers associated to each collaborator. In the following, we describe the *execution and monitoring* stage in Fig. 2.

A *trigger* is a model for an entity to interact with (send transactions to) **SC_EM**. It requires a private key associated to entity’s wallet. In our proposal, a trigger is of three forms: a supervisor trigger, already discussed in the previous section, that configures **SC_EM**; a collaborator trigger, executed is in the side of the IOBP’s collaborator logic to interact with **SC_EM** during the execution and monitoring of the IOBP; a process mining trigger, that retrieve the event log on-chain for executing process mining tasks. In this work, a trigger connects to an Ethereum Blockchain through a wrapper in Java language created with the Web3j library. By providing a wrapper in a portable language as Java, it is more easy for the collaborators (organizations) to adequate their information systems and interact with Blockchain.

Any collaborator, through its trigger, can monitor the IOBP execution by monitoring the status of each task registered in **SC_EM**. This is done by calling the **SC_EM**’s function named *getTaskById(…)*. When called, that function returns:

-status: a boolean value (completed/not completed)
-task name
-task owner (public key of collaborator in charge of the task)
-task type (activity, gate)
-requirements (list of adjacent incoming tasks)
-condition (if the task has a XOR gate requirement)

When an organization/collaborator completes a task, it informs this event to **SC_EM** by calling the function *setTaskOnCompleted(…)*. In this call, the organization (the trigger) sends to **SC_EM** all the information of the event which is later registered, on-chain, in the event log only if the event passes the conformance test. Such test consists in two verifications: (1) the collaborator is the owner of the task being executed and (2) all the tasks being the requirements of the task have been already registered as completed.

### Event log collection

The main input data for process mining algorithms is the event log. In this work, we take advantage of deployed **SC_EM** to collect the events for each IOBP instance execution. This has not been considered in previous works. All the events sent by collaborators during the execution of one IOBP instance form a trace, which is registered on-chain. The set of all traces is the event log. By having the event log on-chain, that information is reliable for any collaborator to run process mining algorithms.

In our approach, the same **SC_EM** is used to monitor the execution of each IOBP instance. Other works as Sturm et al. (2019) use one smart contract instance per IOBP instance. That approach is costlier because a new smart contract instance must be deployed in Blockchain for each new IOBP instance. Furthermore, collecting the traces from each instance implies a more elaborated control. In our approach, we create the **SC_ELC** smart contract only to process all the logic to create the event log. Both, **SC_EM** and **SC_ELC** are linked. All the IOBP instances are registered in a mapping structure in **SC_EM** called *executionInstances*. An instance in execution has an associated trace, which keeps information regarding the execution status of all the tasks in the IOBP. Note that this trace in **SC_EM** is associated to a trace in **SC_ELC**, which keeps information related to the events for each task already executed.

**SC_EM** contains a structure to model a trace with the attributes: caseId (instance number), gcondition (variable to manage conditions in XOR gates), and states (task completed or not completed-required for the conformance test). Once **SC_EM** is configured, a new IOBP instance starts every time the first task in the IOBP is executed, which at the time initiates a new trace in **SC_EM** and in **SC_ELC**. Each next executed task will produce a new entry in the corresponding trace in **SC_EM**, but not necessary registered in **SC_ELC** because the events coming from the triggers are cleaned before registered in the event log, and it is possible that some of these events will be discarded (see more details in next section).

When a collaborator completes a task, it informs this fact to **SC_EM** by invoking the function *setTaskOnCompleted(…)*. The event information includes: (1) caseId, (2) taskId, (3) taskName, (4) timestamp and (5) token. **SC_EM** uses (2) and (5) to execute the conformance test and updates the condition variable if needed. Together with this information from the collaborator, **SC_EM** and **SC_ELC** add information to the event that is later inserted in the trace associated to the process instance. Table 3 shows a summary of data registered in a trace and its source. As it can be observed, Case ID, Activity name and Event timestamp are taken from data sent by the triggers; Event ID is provided by **SC_ELC**, and Event cost and Event resource are provided by **SC_EM**. All these data is recorded in the event log by calling the function *recordEvent(…)* of **SC_ELC**.

**Table 3:** Event data from *Triggers* and SC_EM for event log collection.

Event log attribute	Data field	Source	
Case ID	caseId	Trigger	
Event ID	EventId		**SC_ELC**
Activity name	taskName	Trigger	
Event timestamp	timestamp	Trigger	
Event cost	tx.gasprice		**SC_EM**
Event resource	msg.sender		**SC_EM**

### Event log data cleaning

Events coming from collaborator triggers usually contain errors (duplicate, incomplete or inconsistent data). This implies data quality problems that affect the quality and reliability of process models discovered with process mining algorithms. These problems are due to the fact that data sent by triggers may not be automated and errors could be injected by humans operating the information systems of collaborators in the IOBP. Another cause is that triggers run in different platforms and data could have different formats. Thus, pre-processing and data cleaning are necessary. In this work, data quality in the event log is approached by pre-processing the event data originated in **SC_EM** by a specific smart contract called **SC_PDC** prior to be registered by **SC_ELC** in a trace and hence in the event log.

**SC_PDC** analyzes the data described in Table 3 and sent by **SC_EM** to verify if any of the next problems is present:

1. Missing data: each event must contain Case ID, Activity and Timestamp. If any of these fields is missing, the event is considered as incomplete. The solution is removing the event.
2. Duplicated events, *e.g*., two events has the same name and the same timestamp. If a given event already exists in a trace in the event log, that incoming duplicated event is discarded.
3. Unanchored event: it occurs when the format for the timestamp field provided by triggers is different. The expected format for the timestamp field is *MM/dd/yyyy HH:mm:ss*. Any data for this field in another format is manipulated to met the specification.
4. Form-based event capture: this situation occurs when data from a form (web) are send by the trigger and considered as several events. That will lead to interpret all those events as parallel tasks in the process model. To approach this problem, the trace is explored to verify the presence of events with the same resource and timestamp. The resource field is also verified as an organization can be using a (web) form to register the IOBP events.

Algorithm 2 describes the pseudocode of *setTaskOnCompleted(…)*, which is the main function in **SC_EM** where IOBP execution is orchestrated, events from collaborator triggers are formed and sent to **SC_PDC** for pre-processing and cleaning, and finally cleaned/repaired events are registered by **SC_ELC** in the event log.

**Algorithm 2:** SetTaskOnCompleted (SC EM).

**Require:** *event* = {*caseId, taskId, taskName, timestamp, token*}
**Ensure:** *bool*: ‘*true*’ if the task is correctly registered. Otherwise, ‘*false*’.
1: checked ←*conformanceTest(msg.sender, event)*
2: **if** !checked **then**
3: **return** *false*
4: **end if**
5: *executionInstances*[*caseId*].states[*taskId*] ←*true* $\triangleright$ task marked as completed in the SC since it passes the conformance test
6: **if** *event.taskId*.type == START_EVENT **then**
7: createNewExecutionInstance(*caseId*)
8: **SC_ELC**.newTrace(*caseId*)
9: **end if**
10: **if** *event.taskId*.type == TASK **then**
11: scEvent← new SCEvent()
12: scEvent.add(*event.caseId, event.taskName, event.timestamp*)
13: **SC_ELC**.testRemove(*scEvent*)
14: **if** *scEvent* was removed **then** $\triangleright$ Event is duplicated
15: return *false*
16: **end if**
17: scEventCleaned← **SC_PDC**.preProcessAndClean(*scEvent*)
18: scEventCleaned.add(*eventId*, *event*.taskName, *event*.timestamp)
19: scEventCleaned.add(*msg*.sender, *tx*.gasprice)
20: **SC_ELC**.recordEvent(*caseId*, scEventCleaned) $\triangleright$ new execution data added to the event log
21: **end if**
22: **return** *true*

In Algorithm 2, an event is received containing useful information related to the task that is intended to be registered as executed. First, a test of conformance is done to ensure the task has its prerequisites completed and the event’s source is the executor of that task (line 1). If the test fails, the Blockchain negates the registration (line 3). If the test succeeds, then the event is processed to be registered in the event log (lines 5 to 22). In this case, the event could generate a new trace in the event log (line 7) if it is the start task in the business process. The event is removed if it is a duplicated event (line 14). If not, data is first cleaned and then registered in the event log (lines 17 to 20).

**Figure 4:**
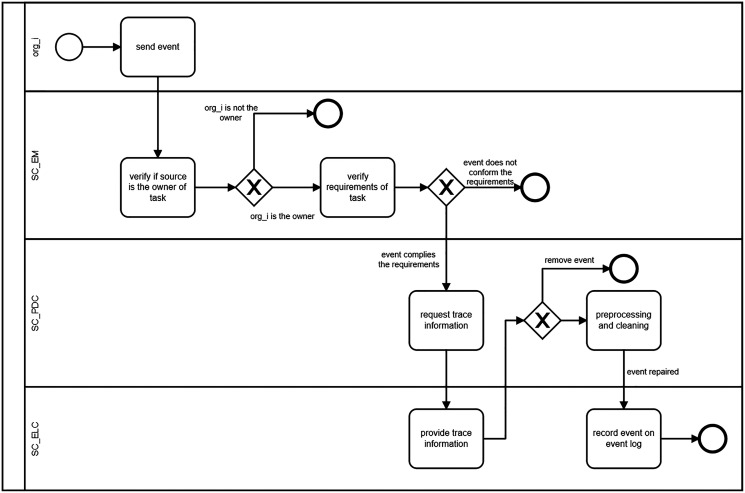
Collaboration among the three smart contracts for IOBP execution monitoring, pre-processing and data cleaning of events and event log collection.

As a summary, Fig. 4 shown a collaboration diagram among a collaborator trigger and the three smart contracts proposed in this work to manage the execution, monitoring, and IOBPs mining (stages 1 and 2 in Fig. 2).

### Event log formatting

Stage 3 in Fig. 2 comprises *event log formatting* and its retrieval for process mining. An entity with the proper permissions in the application domain (process mining trigger) can request the event log to **SC_ELC** and use it to obtain the model of real execution of the documented IOBP by means of a process mining tool. Event log formatting is required because most of process mining tools require the event log to be in a specific format. The most common are the XES (eXtensible Event Stream) and the CSV (Comma Separated Values) formats. For that reason, **SC_ELC** includes functions to retrieve events and traces from the event log. These data is then transformed (off-chain) to the proper format. First, events and traces are converted to CSV and then, event and traces are transformed from CSV to XES.

CSV format is obtained by accessing each event in the event log through the **SC_ELC**’s functions *getNumberOfTraces(), getEventsCount(caseId)*, and *getEvent(caseId, eventId)*, which return the number of traces, number of events in a trace and event in a trace, respectively. XES format is obtained from a translator based on the OpenXES library. A XTrace object is created for each trace in the CSV-formatted event log, and each event in that trace is converted to a XEvent, by adding the attributes needed as XAttributeMap objects. XTrace objects are added to an XLog object that collects the event log in XES format. Finally, the XLog object is serialized by using the Spex library to generate the XES file.

Finally, stage 4 in Fig. 2 deals with using a process mining tool to obtain the IOBP model from the event log collected on-chain. This part is flexible in the sense that once the event log is available in the proper format, any available process mining tool can be used.

## Experimental evaluation and results

The method presented in this paper allows trust among the collaborators in an IOBP at the time it collects and prepares the event log required in process mining tasks. For both purposes, the method relies on Blockchain and smart contract technologies. We assessed the feasibility of this method, depicted in Fig. 2, by deploying it as a software tool written in Java and available in https://github.com/amolina-97/IOBPBC/tree/master/Simulator%20project.

### Tools and setup

**Figure 5:**
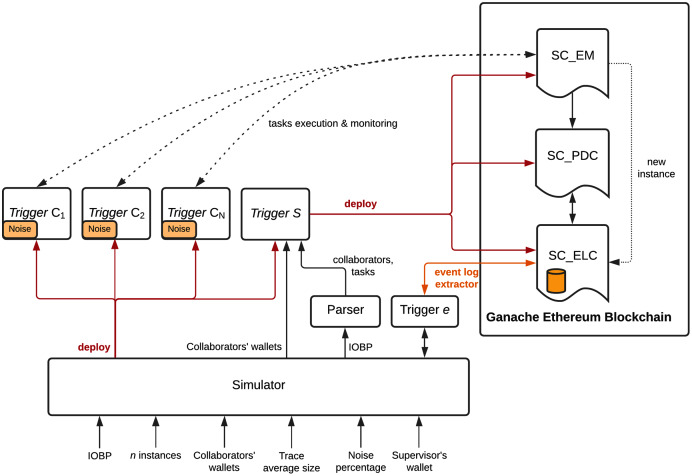
Solution deployed. IOBP execution by threaded triggers. Event log collection with datacleaning, on-chain.

Parser being part of our method uses the Camunda library to process IOBP models in XML BPMN 2. The three smart contracts proposed in this work and discussed in the previous section were implemented in Solidity and executed in the Ganache framework for a private Ethereum Blockchain. Triggers were implemented in Java using the Web3j library. Collaborators in an IOBP are clients that connect to Ganache, which manages the internals of the Blockchain that runs the three smart contracts proposed in this work (see Fig. 5).

Equipment for experimentation consisted of two computers Intel Core 2 Duo 3.06 GHz, 16 GB RAM DDR3, 500 GB HDD; and in one computer Intel Core i5 2.66 GHz, 16 GB RAM DDR3, 1 TB HDD. Since the Ganache framework is for a private Blockchain, the entire method was deployed in each computer.

### Data

We use representative IOBPs in the literature for experimental evaluation: Supply chain (Weber et al., 2016), Incident management (Mühlberger et al., 2019), and Rehabilitation (Köpke, Franceschetti & Eder, 2019). Table 4 shows main characteristics of these IOBPs.

**Table 4:** IOBPs and their characteristics for experimental data.

IOBP	Tasks	Gateways
Supply chain (Weber et al., 2016)	10	2
Rehabilitation (Köpke, Franceschetti & Eder, 2019)	9	4
Incident management (Mühlberger et al., 2019)	9	6

### Validation

For each IOBP, we executed our method that covers: (*i*) deploying the IOBP specified in BPMN in the Blockchain; (*ii*) simulating the IOBP execution by modeling collaborators as execution threads that interact with the smart contracts in the Blockchain; (*iii*) collecting the event log during IOBP’s execution, including data cleaning in that process; (*iv*) preparing data in the event log for process discovery; and (*v*) executing the process discovery task. Collaborators are orchestrated by **SC_EM** and events generated by triggers during an IOBP instance execution are processed by **SC_EM**, **SC_PDC**, and **SC_ELC**.

Simulation is required to validate the complete method which deploys the interorganizational business process (IOBP) in the Blockchain, orchestrates its execution, collects the data at the time the process is executed and prepared the event data for process mining tasks. Without simulation, such validation would be difficult, as the IOBP should be implemented in real scenarios (organizations supported by information systems), and many real executions should be required to generate an event log with the amount of traces usually used in process mining tasks (above 1,000). Obtaining this amount of traces for the three IOBPs considered for validation, without simulation, would be unfeasible. The simulator and each of the components for deploying our proposed method is shown in Fig. 5.

The simulator receives as input the IOBP in BPMN notation, the wallets of collaborators and supervisor, the number of traces to simulate, and two parameters to induce noise in the events. For a real implementation, only the three first parameters are needed for the software tool. The traces collected in the Blockchain (on-chain) and available as an event log can be requested at any moment. Since the event log is in the Blockchain, the access to it is also through a trigger.

### Metrics

Models discovered from the event log created, on-chain, are evaluated using the fitness metric defined by Naderifar, Sahran & Shukur (2019). This metric quantifies the capacity of discovered model (as-is) to express the behavior registered in the event log (to-be). We also use the metric precision (Adriansyah et al., 2013), which quantifies the allowed behavior by the discovered model that is not present in the event log. A model with low precision is the one that allows behavior never ‘seen’ in the event log. Fitness and precision are in the range [0, 1], being one the optimum. With these two metrics, we also evaluate the discovered models using the F-measure metric (Marin-Castro & Tello-Leal, 2021) computed as in Eq. (1).

(1) $$Fmeasure = 2*\displaystyle{{Precision \times Fitness} \over {Precision + Fitness}}$$

In the experimentation, we also evaluated the impact of using data cleaning as part of the event log collection mechanism. It has been suggested from the literature that data cleaning impacts positively in the results obtained when executing process mining tasks, in our case, the process discovery task under metrics of fitness, precision and F-measure. To this last end, we used two tools available in the literature: ProM and P-miner.

### Noise in event data

In each test that includes all the stages described in Section Validation, we included a mechanism to also simulate the presence of noise in events created and sent by collaborator triggers to **SC_EM**. This is done to simulate a more realistic execution of the IOBP. For a given event, the introduced noise is one of four different types (the most common reported in (Suriadi et al., 2017)):

1. Missing values: one or more empty values are in the event.
2. Unanchored event: timestamp granularity is modified (data or time or both can be altered. The format can also be modified (the expected format is ‘MM/dd/yyyy HH:mm:s’).
3. Duplicated events: the event is replicated.
4. Form-based event capture: the timestamp of the event is the same as other already registered.

Noise is randomly inserted, affecting a number or events no greater that 50% the average size of a trace.

### Results

We ran three experiments to evaluate the proposed solution depicted in Fig. 1. For each experiment, we used the three IOPBs described in previous sections. The experiment considered the running of the entire method, from IOBP deployment in the Blockchain until the execution of process discovery over the collected data obtained from the Blockchain. In the first experiment, no noise is injected, which corresponds to the ideal case where each organization in the IOBP performs as expected and executes the business process as documented. This is unrealistic because the real executed process generally is far similar to the documented one: tasks can be interchanged, cycles can be present, or tasks cloud be omitted. In the second experiment, noise was injected to simulate the real operations of IOBPs and the pre-proccessing and data cleaning algorithm was enabled to correct errors in the execution data. Thus, the data in the event log was improved at a degree later evaluated in the process discovery task. In the third experiment, noise was simulated but the pre-proccessing and data cleaning algorithm was disabled.

Experiments two and three allowed to study the impact of pre-processing and data cleaning in the event log, in the context of process discovery. The simulator was programmed to inject noise (missing values, unanchored event, duplicate events and form-based event capture) in the collaborator triggers in 10%, 20% and 30% of traces generated during IOBP execution.

Table 5 shows the details of event logs collected during the execution of the three IOBPs orchestrated by **SC_EM**. In all cases, a total of 1,200 traces were registered by **SC_ELC**, each containing repaired data by the **SC_PDC** (indicated in the row labeled as ‘LD on’). As a reference, and in order to show the impact of data cleaning, Table 5 also includes statistics of the event logs for the case when no data cleaning is done (indicated in the row labeled as ‘LD off’) and in the ideal, unfeasible, perfect case of no noise present (indicated in the row labeled as ‘No noise’).

**Table 5:** Event log statistics for the three IOBPs used in experimentation. Data includes the case when no noise is inserted. If noise is inserted, data can be cleaned, on-chain, or not cleaned.

Case	Experiment	Noise %	Traces	Events	Different events	Different traces	Trace size average
No noise	Incident management	0	1,200	6,446	6,446	3	5.36
	Rehabilitation	0	1,200	9,600	9,600	5	7.99
	Supply chain	0	1,200	12,000	12,000	2	9.99
LD on	Incident management	10	1,200	6,204	6,204	24	5.16
		20	1,200	6,024	6,024	38	5.01
		30	1,200	6,032	6,032	46	5.02
	Rehabilitation	10	1,200	9,054	9,054	39	7.53
		20	1,200	9,016	9,016	56	7.50
		30	1,200	8,888	8,888	70	7.40
	Supply chain	10	1,200	10,104	10,104	68	8.41
		20	1,200	10,173	10,173	89	8.47
		30	1,200	10,125	10,125	105	8.43
LD off	Incident management	10	1,200	6,694	6,607	93	5.57
		20	1,200	6,971	6,782	184	5.80
		30	1,200	6,829	6,572	265	5.68
	Rehabilitation	10	1,200	9,744	9,616	125	8.11
		20	1,200	9,962	9,678	244	8.29
		30	1,200	10,132	9,697	354	8.43
	Supply chain	10	1,200	12,254	12,064	122	10.20
		20	1,200	12,469	12,102	241	10.38
		30	1,200	12,677	12,163	359	10.55

The ideal case of no errors in event data is unfeasible as data from triggers are generally sent by humans operating the information systems, different platforms can be used and situation out of control are present (hardware fails, connection delays, system halts, for example). In the ideal case (no noise) the number of events and the size of a trace are the reference values. Contrary, when errors are present and not repaired, the number of different traces increases considerably, leading possibly to inconsistent models difficult to analyze (spaghetti-like models). These problems are mitigated and reduced by the cleaning strategy, which maintains the number of different traces and average size trace closer to the reference values.

**Figure 6:**
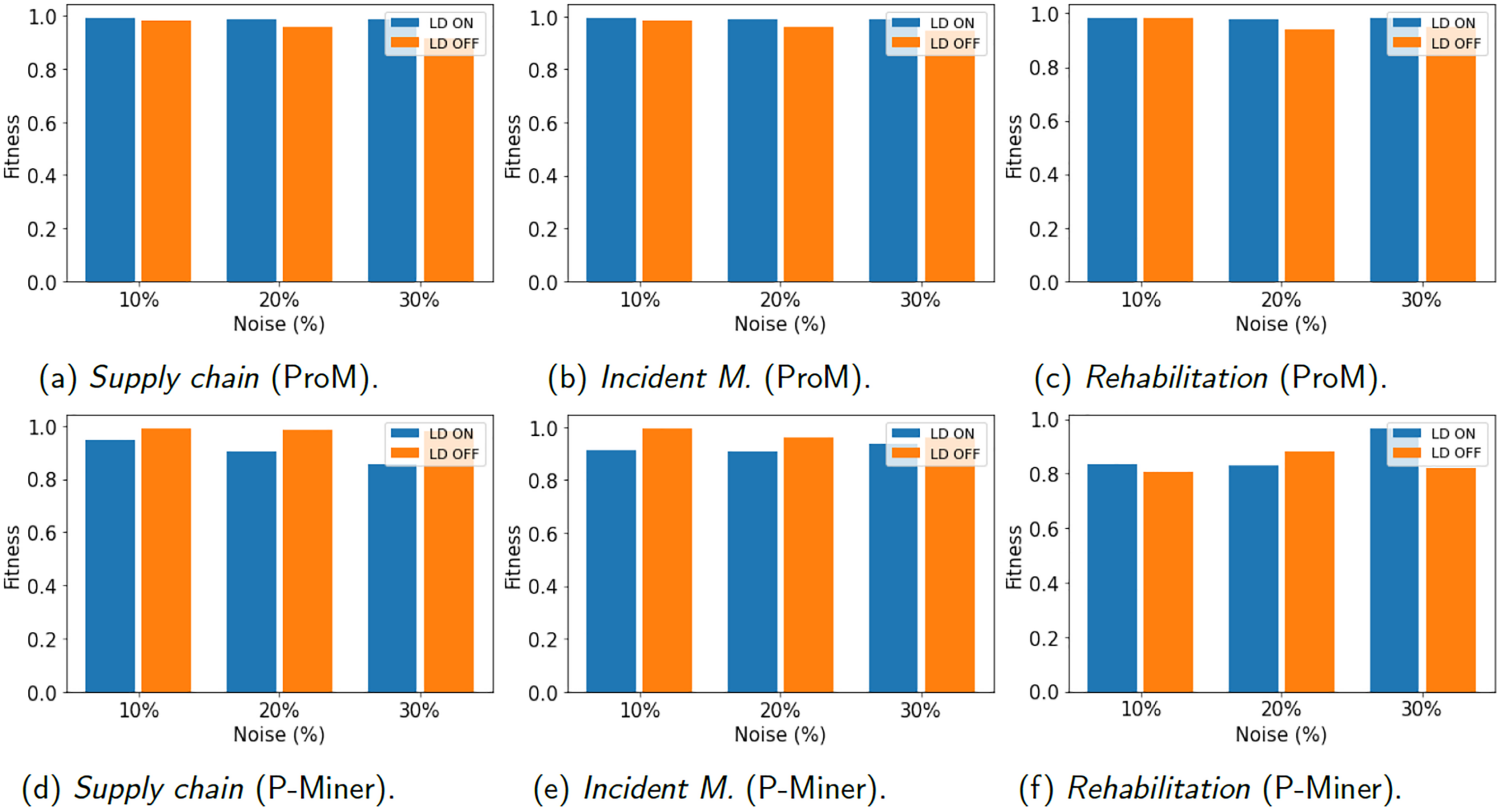
(A–F) Models evaluation (Fitness) using 1,200 traces.

**Figure 7:**
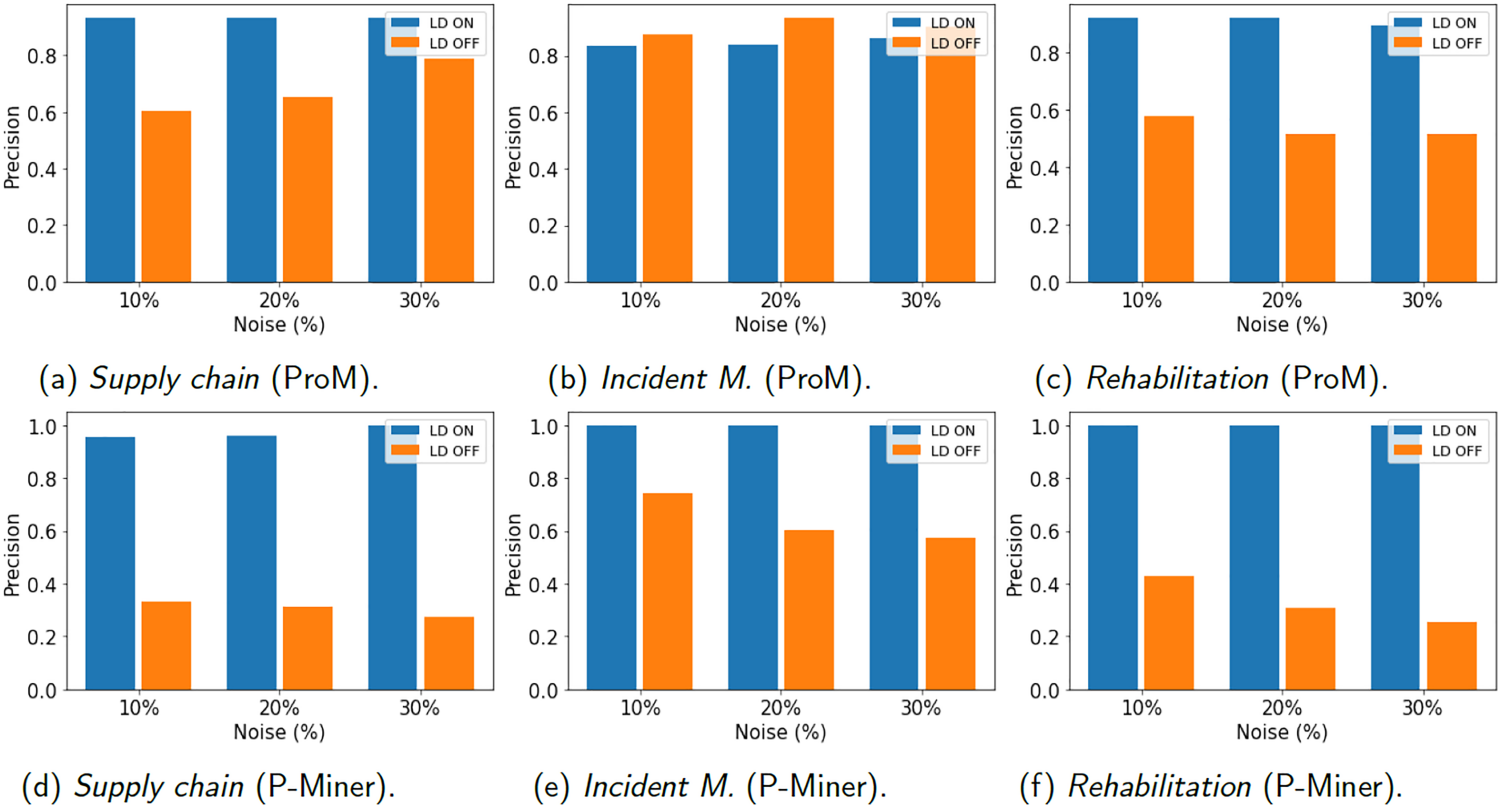
(A–F) Models evaluation (Precision) using 1,200 traces.

**Figure 8:**
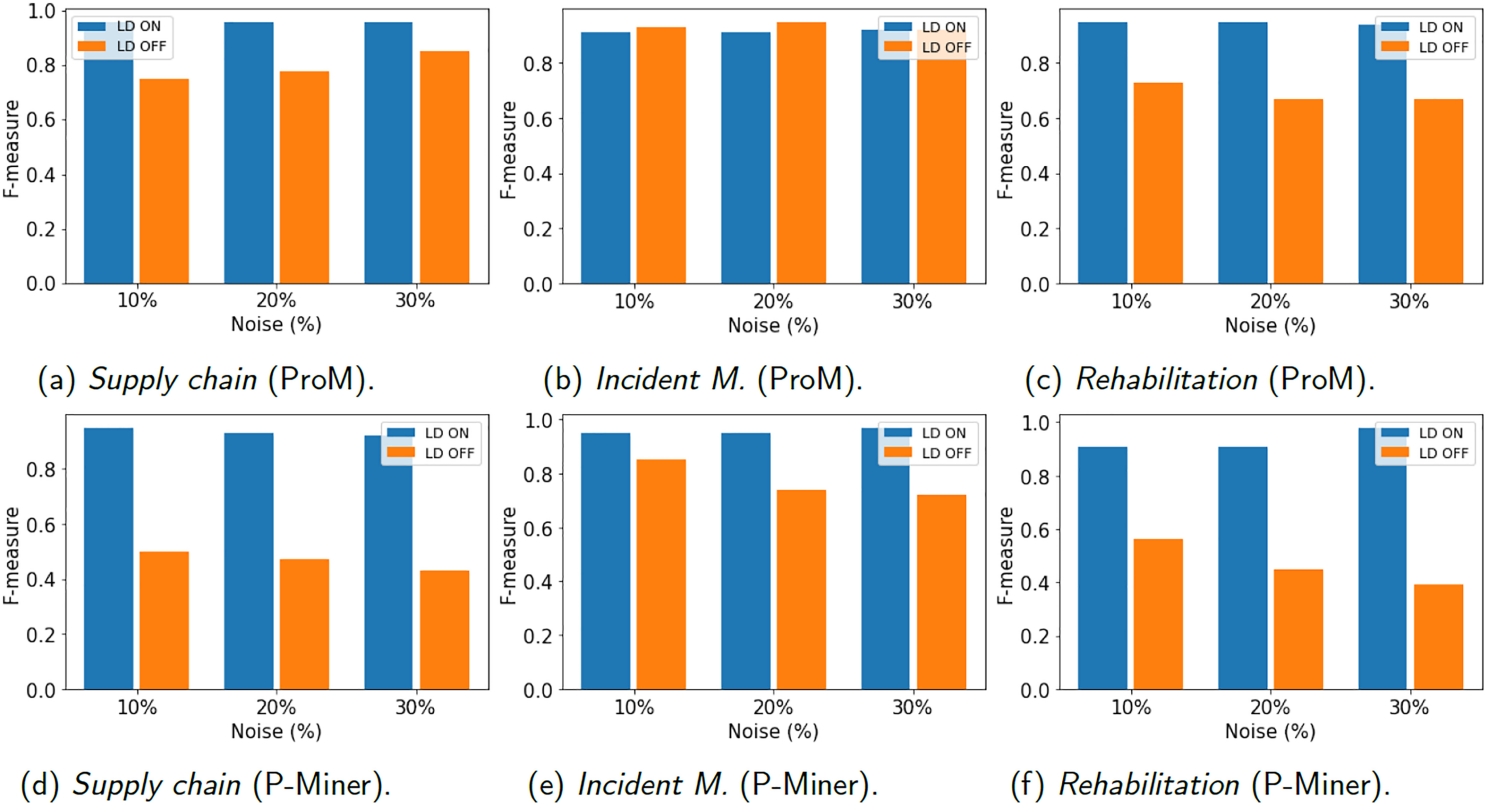
(A–F) Models evaluation (F-measure) using 1,200 traces.

Figures 6–8 show results for the metrics of fitness, precision and F-measure respectively, computed with ProM and P-Miner. In the case of ProM, we used the plug-ins Mine Petri Net with Inductive Miner for the model discovery, and Replay a Log on Petri Net for Conformance Analysis for fitness and Check Precision based on Align-ETConformance for precision. In the case of P-Miner, the tool computes fitness, precision and F-measure automatically when the model is discovered.

Figure 6 reveals that fitness is not affected in general by errors in event data, that is, data cleaning does not significantly improve the fitness in the discovered models. That is, fitness values are high without repairing data in the event log, and the differences in fitness between the models discovered from the cleaned logs are minor, and close to the fitness of the uncleaned logs. Thus, the discovered models have a good fit with the documented models.

However, Fig. 7 reveals that precision is greatly affected when no data cleaning is activated, and it deteriorates when the percentage of induced noise is greater. On the contrary, if data cleaning is used, the models keep a precision near to one (the best) even at the presence of noise. One of the factors that possibly influences in this fact is that filtering and repairing data from the event log lead to contain less behavior than the original event log. Therefore, precision is affected if a process model supports too much behavior, that is, precision is lower for the event log whose data has not been repaired.

A special case is for the precision of Incident Management computed by ProM. In this case, the precision improves when no data cleaning is used and when the percentage of noise increases. This can be possible due to the fact that erroneous data produce alternative running paths that are still acceptable for the model. In general, precision improves due to the data cleaning. Figure 8 shows the mean performance between fitness and precision (F-measure) obtained by the models, in which it can be observed that the low precision percentage obtained in the cases when no data cleaning is activated has a significant impact.

However, it is possible to identify that when data cleaning is used, the process model maintains F-measure between 0.9 and 1. Therefore, it can be established that the F-measure presented by the process models is acceptable and reflects what is observed in the event log without being so general.

## Conclusion

This paper presented, for the first time, a practical approach that uses Blockchain and smart contracts technologies for supporting inter-organizational business process (IOBP) execution management at the time that collects the event log required for IOPB mining. The approach included dynamic data cleaning on-chain to remove errors in data events from collaborators that could affect the discovered models by process mining tools. Blockchain ensures trust during the IOBP execution while the event log collection on-chain provides confident data for process mining. Our method was completely implemented and validated over three IOBPs, which demonstrates its practicality for real scenarios.

By using three smart contracts, our proposal can deploy any IOBP in BPM notation in a Blockchain, ensuring the business logic when executed even in the presence of untrusted collaborators and later providing the event log in the proper format for process mining tasks. We provide to the community a software tool that implements the proposed approach, including the three smart contracts for execution, data cleaning and event log collection. Both, the method and the software tool, are the main contributions of this work.

Future work consists in improving the smart contract for pre-processing and data cleaning. Currently, only the most common problems in event data are considered and data cleaning uses information only from the trace itself. A second data cleaning pass on the entire event log can be applied prior to process discovery, for example, to correct order of events in traces or to filter infrequent or chaotic activities. Furthermore, future directions consider analyzing the complexity of algorithms to bound the performance of proposed method and evaluate the viability of implementing run-time acceleration strategies.

## Supplemental Information

10.7717/peerj-cs.731/supp-1 Supplemental Information 1 Simulator. URL of software tool

## Additional Information and Declarations

### Data Availability

The following information was supplied regarding data availability: The data is available at GitHub: https://github.com/amolina-97/IOBPBC. The simulator is available at GitHub: https://github.com/amolina-97/IOBPBC/tree/master/Simulator%20project.
